# Application of response surface methodology for optimization of natural organic matter degradation by UV/H_2_O_2_ advanced oxidation process

**DOI:** 10.1186/2052-336X-12-67

**Published:** 2014-04-15

**Authors:** Reza Rezaee, Afshin Maleki, Ali Jafari, Sajad Mazloomi, Yahya Zandsalimi, Amir H Mahvi

**Affiliations:** 1 Department of Environmental Health Engineering, School of Public Health, Tehran University of Medical Sciences, Tehran, Iran; 2 Kurdistan Environmental Health Research Center, Kurdistan University of Medical Sciences, Sanandaj, Iran; 3 Center for Solid Waste Research, Institute for Environmental Research, Tehran University of Medical Sciences, Tehran, Iran; 4 National Institute of Health Research, Tehran University of Medical Sciences, Tehran, Iran

**Keywords:** Natural organic matter, Total organic carbon, UV/H_2_O_2_, Response surface methodology

## Abstract

**Background:**

In this research, the removal of natural organic matter from aqueous solutions using advanced oxidation processes (UV/H_2_O_2_) was evaluated. Therefore, the response surface methodology and Box-Behnken design matrix were employed to design the experiments and to determine the optimal conditions. The effects of various parameters such as initial concentration of H_2_O_2_ (100–180 mg/L), pH (3–11), time (10–30 min) and initial total organic carbon (TOC) concentration (4–10 mg/L) were studied.

**Results:**

Analysis of variance (ANOVA), revealed a good agreement between experimental data and proposed quadratic polynomial model (R^2^ = 0.98). Experimental results showed that with increasing H_2_O_2_ concentration, time and decreasing in initial TOC concentration, TOC removal efficiency was increased. Neutral and nearly acidic pH values also improved the TOC removal. Accordingly, the TOC removal efficiency of 78.02% in terms of the independent variables including H_2_O_2_ concentration (100 mg/L), pH (6.12), time (22.42 min) and initial TOC concentration (4 mg/L) were optimized. Further confirmation tests under optimal conditions showed a 76.50% of TOC removal and confirmed that the model is accordance with the experiments. In addition TOC removal for natural water based on response surface methodology optimum condition was 62.15%.

**Conclusions:**

This study showed that response surface methodology based on Box-Behnken method is a useful tool for optimizing the operating parameters for TOC removal using UV/H_2_O_2_ process.

## Background

Natural organic matters (NOMs) are complex compounds with different molecular composition and size that can be found in surface and ground water resources. Their quantities, specifications and degradation depend on the season, weather, microorganisms activities, human activities and the eutrophication status of water resources [1-3]. The presence of NOM in water treatment processes and distribution systems is undesirable and cause several problems such as biological growth, taste, odor, corrosion and membrane fouling [4-6]. NOMs can be removed via several methods such as coagulation, conventional filtration through different media, enhanced coagulation, membranes, ion exchange,adsorption and advanced oxidation process [5,7,8]. While the removal of NOM in order to reduce the formation of disinfection byproducts is becoming increasingly important, some conventional treatment methods cannot remove NOMs effectively [7,9]. Coagulation process (including the use of iron salts and alum) is effective to remove high molecular weight fraction of hydrophobic NOM, however, it is not effective for the removal of hydrophilic segments.

Although technologies such as membrane process is an effective option, but due to the high cost, it is not widespread in developing countries [5,7]. Advanced oxidation processes (AOPs) are a variety of methods to eliminate or oxidize NOM from raw waters. These process include O_3_/H_2_O_2_, O_3_/UV, UV/H_2_O_2_, TiO_2_/UV, H_2_O_2_/catalyst, Fenton and photo-Fenton processes as well as ultrasound [5,10,11]. In general, advanced oxidation processes, refers to the processes hydroxyl radicals (OH∙) are generated as an intermediate via different methods [12]. Due to highly oxidizing potential of hydroxyl radicals (2.8 v), advanced oxidation processes are often based on production of this active radical. Application of UV radiation and a suitable oxidizing agent such as H_2_O_2_ is one of the most effective methods for advanced oxidation. This method is highly effective for removing NOM and refractory organic compounds from water. In this process, H_2_O_2_ molecule is divided into two hydroxyl radicals because of absorbing photons. These radicals can attack organic molecules under proper operating conditions to produce end products such as water, CO_2_ and inorganic acids [5,11,13]. During treatment with UV/H_2_O_2_, NOM compounds are mostly oxidized and aromatic contents are reduced. Also during the process, high molecular NOM are transformed into low biodegradable compounds such as aldehydes, carboxylic acids [1,14]. Generated hydroxyl radicals, reduces both total organic carbon (TOC) and disinfection by product formation potential (DBPFP) in raw water [1]. It has been reported that at proper concentration of hydrogen peroxide and appropriate UV dose, this process can completely mineralize NOM into inorganic compounds [14].

In most related studies, experiments have been conducted with changing the independent variables and keeping the others constant. In such studies, in addition to large number of runs, spending time and cost, the interactions between the operating variables are not revealed. These restrictions can be eliminate using the response surface methodology (RSM) [15,16]. RSM is a collection of mathematical and statistical techniques applied for developing, improving and optimizing a response affected by a number of process variables. This method has advantages such as fast and reliable data achievement, understandable interaction effects of different parameters and consequently a significant reduction in experimental runs, time and cost [15-17]. RSM have been successfully applied in different process optimization such as chemistry, biochemistry, physics, environmental science, membrane process and AOPs [15,16].

The aim of present work was to optimize UV/H_2_O_2_ operating parameters for natural organic matter degradation from aqueous solutions by using response surface methodology. The effect of main variables including pH, time, H_2_O_2_ and initial TOC concentration were evaluated using experimental data and a predicted model was developed. Finally, based on RSM optimum condition, TOC removal was studied for natural water samples.

## Materials and methods

### Chemicals and UV/H_2_O_2_ system

In this study, humic acid (Sigma-Aldrich, Germany) and Hydrogen peroxide 30% (Merck, Germany) were used. Sodium hydroxide (Merck, Germany) and chloridric acid (Merck, Germany) were used for pH modification. UV/H_2_O_2_ system consisted of a batch reactor made of smooth stainless steel with an effective volume of 2.5 L, 92 cm length and 7.6 cm diameter. A 55-watt low-pressure mercury lamp, ultraviolet radiation source (UV-C) with an intensity of 50 mW/cm^2^ (Arda company, France) was used.

### Preparation of synthetic and raw water

In this research, TOC removal from synthetic and raw water samples were studied. Synthetic samples were prepared by dissolving a known amount of humic acid in deionized water [18]. Raw water samples were taken in July (2012) from Sanandaj water treatment plant (Kurdistan Province, West of Iran). Samples were immediately stored at 4°C prior to use. The average alkalinity of raw water samples was 200 mg/L as CaCO_3_, the pH was 8.15, the turbidity was 3.5 NTU, the TOC was 4.2 mg/L, the dissolved organic carbon was 3.3 mg/L and the absorption coefficient of UV_254_ was 0.098 cm^-1^.

### Batch experimental program & analytical methods

In this study, TOC was measured as the indicator for humic substances [1]. In each experiment, 2.5 L of feed solution with known initial concentration of TOC, H_2_O_2_ and pH value was poured in the batch reactor for UV irradiation. Sampling was conducted at proper intervals for TOC measurement. TOC was analyzed using a TOC analyzer (TOC-VCPH, Shimadzu, Japan). The percentage removal (Y) of TOC was calculated by Equation 1.

(1) $$Y\;\left(\%\right)=100\;\left(C_{0}-C_{f}\right)/C_{0}$$

where, C_o_ is the initial concentration of TOC (mg/L), C_f_ is the final concentration of TOC (mg/L) after a known time.

### Experimental design and data analysis

For determination of independent variables affecting the TOC removal by UV/H_2_O_2_ process and their interaction effects, RSM method based on Box-Behnken design was applied [17,19,20]. For this design, the main variables are H_2_O_2_ initial concentrations (100–180 mg/L), pH (3–11), time (10–30 min) and initial TOC concentrations (4–10 mg/L). Independent variables in the range of -1 and +1 were coded according to classical methods similar to previous studies (Table 1). In RSM for each response a model is defined that can predict the individual and interaction effect of different parameters. General form of a quadratic model for four variables is as Equation 2 [19,21].

(2) $$\begin{array}{l} Y=\beta_{{}^{\circ}}+\beta_{1}X_{1}+\beta_{2}X_{2}+\beta_{3}X_{3}+\beta_{4}X_{4}+\beta_{11}{X_{1}}^{2}+\beta_{22}{X_{2}}^{2}+\beta_{33}{X_{3}}^{2}+\beta_{44}{X_{4}}^{2}\\ \;+\beta_{12}X_{1}X_{2}+\beta_{13}X_{1}X_{3}+\beta_{14}X_{1}X_{4}+\beta_{23}X_{2}X_{3}+\beta_{24}X_{2}X_{4}+\beta_{34}X_{3}X_{4} \end{array}$$

**Table 1:** Independent variables and experimental levels

**Variable**	**Factor**	**Unit**	**Range and level**		
			**Low (-1)**	**Middle (0)**	**High (+1)**
H_2_O_2_ concentration	X_1_	mg/L	100	140	180
pH	X_2_	-	3	7	11
Time	X_3_	min	10	20	30
Initial TOC	X_4_	mg/L	4	7	10

Where Y = predicted response

β_◦_ = constant coefficient

β_1_, β_2_, β_3_ and β_4_ = linear effect coefficients

β_11_, β_22_, β_33_, and β_44_ = quadratic effect coefficients

β_12_,β_13_, β_14_, β_23_, β_24_ and, β_34_ = interaction effect coefficients

X_1_, X_2_, X_3_, and X_4_ = independent variables

Design-expert (Stat-Ease, trial version) was the software used for designing the experiment, statistical analysis (e.g. analysis of variance) and response surface studies. All presented graphs were generated using the software.

## Results and discussion

### Development and evolution of prediction model

The experimental results, the model predictions and the response (Y) using Box-Behnken design matrix for UV/H_2_O_2_ process is presented in Table 2. The results of analysis of variance (ANOVA) for the independent variables are presented in Table 3. The quadratic polynomial model (Equation 3) for the dependent variable (Y) was fitted. Based on the RSM and quadratic polynomial equation, there can be an empirical relationship between the response and the independent variables. The final obtained equation based on the coded factors is as follows.

(3) $$\begin{array}{l} Y=71.50+3.56x_{1}-15.08x_{2}+3.58x_{3}-11.19x_{4}\\ \;-0.75x_{1}x_{2}+2.25x_{1}x_{3}+0.075x_{1}x_{4}+4.00x_{2}x_{4}\\ \;-1.50x_{3}x_{4}+0.{{52x}_{1}}^{2}-16.{{51\;x}_{2}}^{2}-2.{{76\;x}_{3}}^{2}-5.{{47\;x}_{4}}^{2} \end{array}$$

**Table 2:** The Box–Behnken design matrix for variables along with Experimental and predicted values response

**Run order**	**X**_**1**_	**X**_**2**_	**X**_**3**_	**X**_**4**_	**TOC removal efficiency (Y)**	
					**Experimental (%)**	**Predicted (%)**
1	180	7	20	4	84	82
2	180	7	30	7	80	78.65
3	140	3	20	4	78	79.79
4	140	7	10	4	70	69.73
5	140	7	30	10	53	54.15
6	100	7	30	7	69	67.04
7	140	3	20	10	52	49.40
8	140	7	30	4	82	79.54
9	140	7	10	10	47	49.99
10	140	11	10	7	35	33.56
11	180	7	20	10	61	58.99
12	140	7	20	7	70	71.50
13	140	7	20	7	71.5	71.50
14	100	7	20	10	48.5	51.73
15	100	7	10	7	64	64.37
16	100	7	20	4	71.8	74.26
17	100	11	20	7	40	37.62
18	140	11	30	7	38	40.73
19	100	3	20	7	68	66.29
20	140	3	10	7	66	63.73
21	180	3	20	7	72	74
22	140	3	30	7	69	70.89
23	180	11	20	7	41	43.24
24	180	7	10	7	66	66.99
25	140	11	20	4	40	41.62
26	140	11	20	10	30	27.24
27	140	7	20	7	73	71.50

**Table 3:** Analysis of variance (ANOVA) results of quadratic model for TOC removal efficiency (Y)

**Source**	**Sum of squares**	**Df**	**Square mean**	**F value**	**p-value prob > F**	**Remark**
Model	6336.57	14	452.61	46.56	< 0.0001	Significant
X_1_	151.94	1	151.94	15.63	0.0019	
X_2_	2730.08	1	2730.08	280.84	< 0.0001	
X_3_	154.08	1	154.08	15.85	0.0018	
X_4_	1503.04	1	1503.04	154.62	< 0.0001	
X_1_ X_2_	2.25	1	2.25	0.23	0.6391	
X_1_ X_3_	20.25	1	20.25	2.08	0.1745	
X_1_ X_4_	0.023	1	0.023	2.315E-003	0.9624	
X_2_ X_3_	0.000	1	0.000	0.000	1.0000	
X_2_ X_4_	64.00	1	64.00	6.58	0.0247	
X_3_ X_4_	9.00	1	9.00	0.93	0.3549	
X_1_^2^	1.47	1	1.47	0.15	0.7042	
X_2_^2^	1454.20	1	1454.20	149.59	< 0.0001	
X_3_^2^	40.70	1	40.70	4.19	0.0633	
X_4_^2^	159.87	1	159.87	16.45	0.0016	
Residual	116.65	12	9.72			
Lack of fit	112.15	10	11.22	4.98	0.1786	Not significant
Pure error	4.50	2	2.25			
Cor total	6453.22	26				

Note: R^2^ = 0.9819; Adj- R^2^ = 0.9608; C.V% = 5.13 and PRESS = 656.13.

Routinely to assess the adequacy of a model, the coefficient of determination (R^2^) and the lack of fit test is used [22,23]. Coefficient of determination (R^2^) refers to the changes described by the model to the overall changes. Therefore, whatever R^2^ is closer to 1, the power of fitted model is greater to describe the response changes as a function of the independent variables [24].

**Figure 1:**
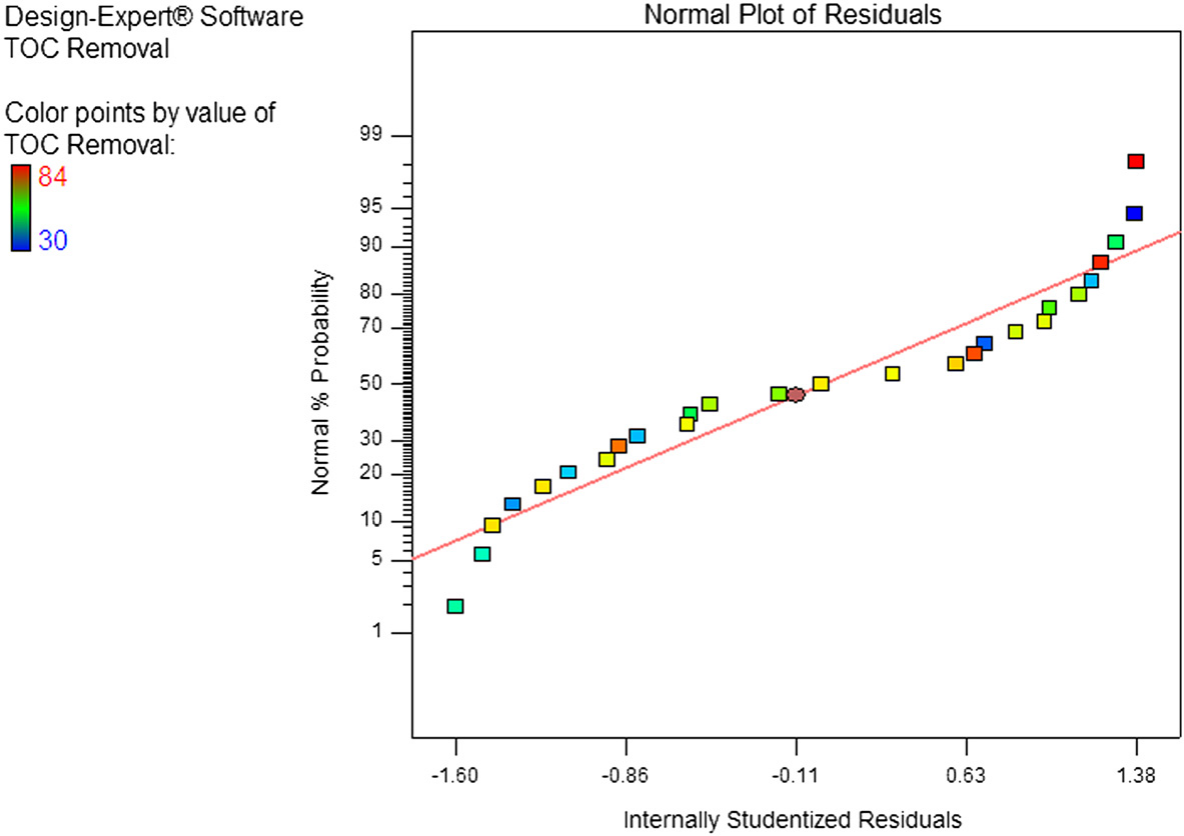
Normal probability plot, TOC removal efficiency (Y).

**Figure 2:**
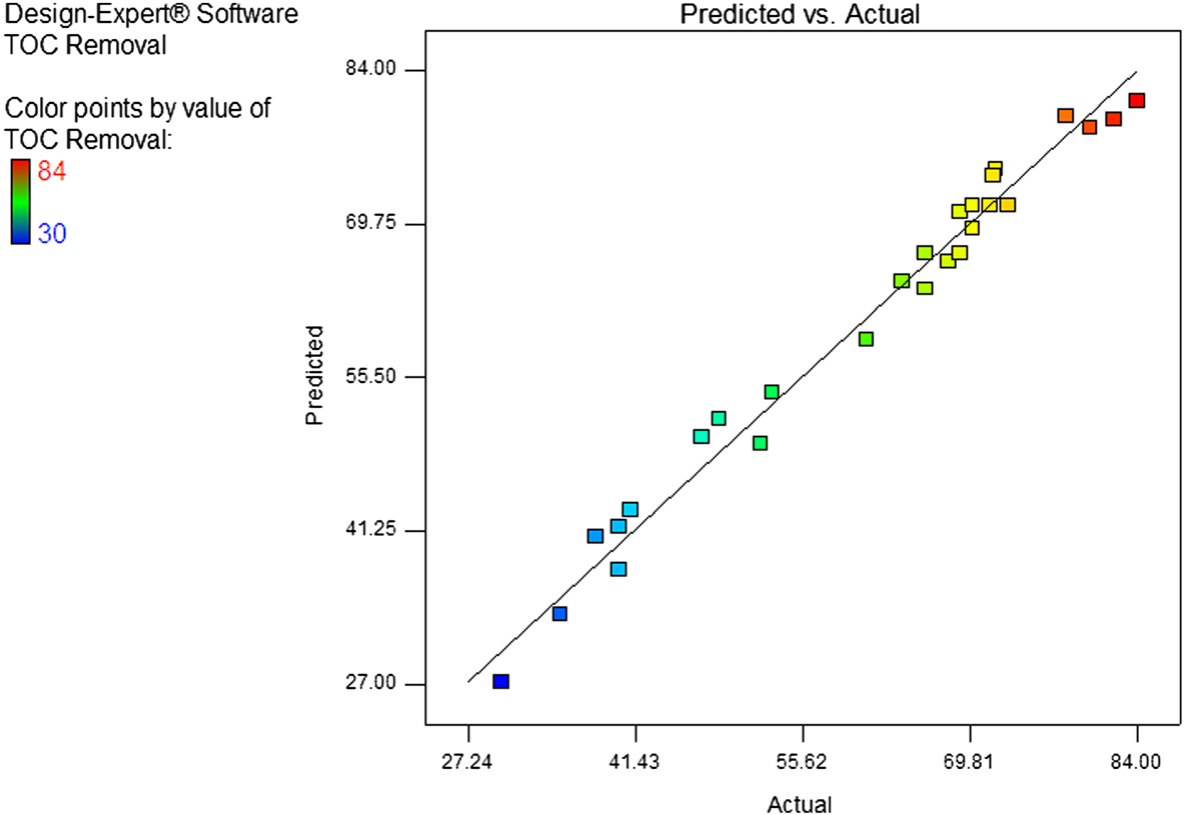
Comparison between the experimental values and the predicted values of RSM model, TOC removal efficiency (Y).

Based on the ANOVA results, R^2^ for the removal of TOC is 0.98 that shows the adequacy of built model. Lack of fit test is a sign of lack of experimental data for a model that the model can not calculate the random errors of experimental data. If the test is statistically significant (p value < 0.005), it indicates that the model is not fit for the prediction of response [22,23]. With regard to Table 3, insignificant value of lack of fit test (p value > 0.05) relative to the pure error indicates that there is good correlation between the variables and process response. Besides determination of coefficient (R^2^), adjusted determination of coefficient (R^2^_adjusted_) is a useful statistical tool to evaluate the model adequacy [15,22,23]. R^2^_adjusted_ is high (0.96) and close to R^2^ value, indicates the adequacy of the developed model to predict the process response (Table 3). According to Table 3, for response (Y), the quadratic model was statistically significant (P <0.0001). Other significant terms of the model are X_*1*_, X_*2*_, X_*3*_, X_*4*_, X_2_X_4_, X_2_^2^, X_4_^2^. Normal probability plot is a graphical method for determining residuals normality [21,25]. A normal probability plot of the residuals versus the response (Y) is presented in Figure 1. Graphical data on the plot located in a position close to a straight line shows that the model sufficiently for TOC removal by UV/H_2_O_2_ process. The experimental and predicted values for Y are shown in Figure 2. Observations indicate a very good correlation between the results obtained by experiments and the values predicted by the statistical model, which shows the success of this model.

### Effect of independent variables on TOC removal

**Figure 3:**
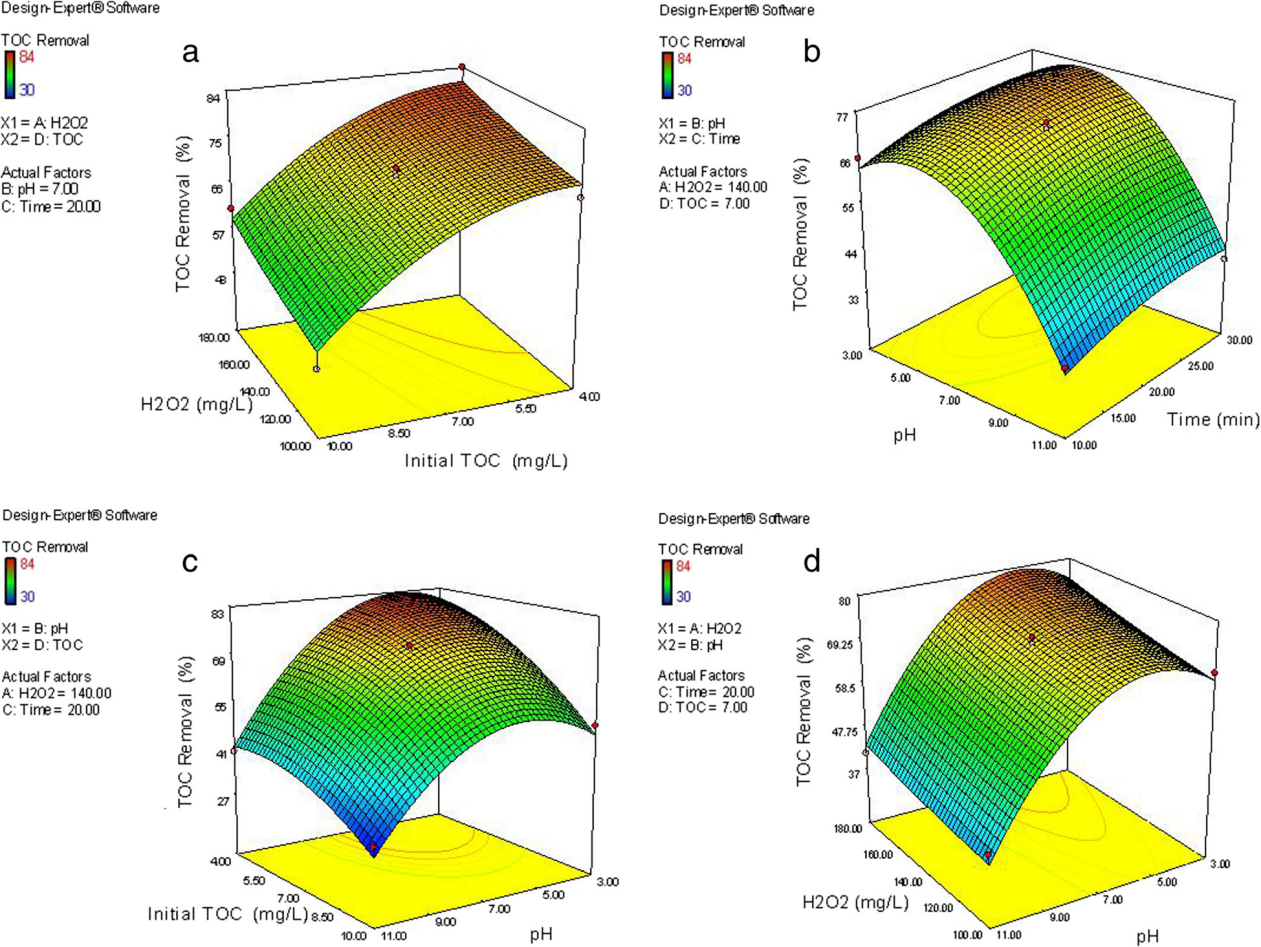
**Three-dimensional response surface plots for TOC removal versus independent variables. (a)** H_2_O_2_ concentration and initial TOC **(b)** pH and time **(c)** Initial TOC and pH (d) H_2_O_2_ concentration and pH.

According to Table 3, the linear effects of four independent variables (initial concentration of TOC, initial hydrogen peroxide concentration, pH of solution and processing time) are significant. Thus, each variable in turn can affect the TOC removal efficiency. P value also shows that the effect of pH and initial concentration of TOC (P <0.0001) is greater than time and H_2_O_2_ concentration (P <0.001). Interaction and second-degree effects of pH and initial TOC concentration are significant, confirming the influence of these two parameters. The effect of the independent variables on TOC removal efficiency as a three-dimensional response surface plot is shown in Figure 3. From Figure 3a and c, by decreasing the initial concentration of TOC (from 10 to 4 mg/L) the removal efficiency increases. Maximum TOC removal was achieved at the lowest initial TOC concentration of 4 mg/L. Generally, UV radiation and hydrogen peroxide produces free radicals such as hydroxyl radicals [1,26,27]. Increasing in initial concentration of humic substances generally decreases the process efficiency, probably because of competition between metabolites of humic substances to react with the hydroxyl radicals as a non-selective agent. Therefore, increasing the initial concentration of humic substances, resulting in increased concentrations of intermediate products in the environment, consequently a significant amount of hydroxyl radicals for the degradation of intermediate products is used [27,28]. NOM can also compete with H_2_O_2_ for reacting with UV radiation. Thus, low concentrations of NOMs allow more H_2_O_2_ photolysis and consequently more hydroxyl radicals production [26]. As it is shown in Figure 3a and d, TOC removal efficiency increased with increasing H_2_O_2_ concentration. in the concentrations range of H_2_O_2_ (100 to 180 mg/L), at low concentration of TOC (4 mg/L), with increasing concentrations of hydrogen peroxide, removal efficiency increased from about 74% to 82%. Oxidation rate increases with increasing H_2_O_2_ concentrations, This is due to the fact that high concentrations of H_2_O_2_ and more absorption of UV rays, increases hydroxyl radical production [27]. Based on the results of similar studies, with increasing concentrations of hydrogen peroxide, oxidation efficiency increases to a certain extent, but in case of excessive concentrations of H_2_O_2_, little effect and even in some cases loss oxidation rate have been reported. Probably high concentrations of hydrogen peroxide act as radical scavenging agent and reduce the amount of active radicals [13,29]. Furthermore, an increase in H_2_O_2_ concentration results in its direct reaction with hydroxyl radicals and HO_2_^°^ production with lower oxidation capacity than OH^°^[27]. Although the concentration of hydrogen peroxide plays an important role in the oxidation process, but in high concentrations due to scavenging the hydroxyl radicals, it is less effective [5]. TOC removal efficiency increases with the increase of solution pH from 3 to 7 (Figure 3b, c, d), And then from pH, 7 to 11 TOC removal efficiency decreases. Therefore, the maximum TOC removal efficiency was achieved at neutral pH. Initial pH of a solution plays an important role in the oxidation process. Hydrogen peroxide is stable in the pH range of 5–9, the more free radicals (HO_2_∙, OH∙) can be formed by UV photolysis. Presence of bicarbonate and carbonate alkalinity (especially carbonate alkalinity) can compete with NOM for scavenging produced hydroxyl radicals, resulting in decrease of NOM removal at alkaline pH values [1,10,26]. The effect of pH on UV/H_2_O_2_ process depends on the nature of the contaminants. For example it has been reported that the decomposition of phenol by photochemical process is more effective at acidic pH values [30,31].

With increasing processing time from 10 to 30 min, removal of TOC increased and the maximum removal efficiency was obtained at 30 min (Figure 3b). In fact, with increasing reaction time, hydrogen peroxide has more time to react with UV, resulting in the production of more hydroxyl radicals [7,27,28]. It was revealed that the effects of pH and initial TOC concentration were more significant than H_2_O_2_ concentration and processing time.

### Process optimization and evaluation

For process optimization, the maximum TOC removal efficiency was optimized at minimum consumption of H_2_O_2_. Optimum values of process variables and test results under optimal condition are presented in Table 4. Accordingly, TOC removal efficiency of 78 .02% was determined at the optimum conditions of independent variables. The maximum TOC removal efficiency using confirmation experiments was 76.50%. Therefore, there is good agreement between the predicted and experimental affirmation results at optimum condition that confirms the developed model. It is concluded that RSM is a powerful tool to determine the exact values of the independent variables [32].

**Table 4:** Optimum value of the process variables for maximum TOC removal efficiency and Predicted and experimental value for the responses at optimum conditions

**H**_**2**_**O**_**2 **_**concentration (mg/L)**	**pH**	**Time (min)**	**Initial TOC (mg/L)**	**TOC removal (%)**	
				**Predicted**	**Experimental**
100	6.12	22.42	4	78.02	76.50

### Treatment raw water samples under optimal conditions

In this phase of the study, the TOC removal efficiency using UV/H_2_O_2_ process for natural raw water samples was conducted under optimal conditions. Based on the values obtained for the optimal concentration of hydrogen peroxide (100 mg/L), time (22.4 m) and normal raw water quality (TOC = 4.20 mg/L, pH = 8.15) . The results revealed that TOC removal under optimum conditions for natural water samples decreased to 62.15% comparison with synthetic samples. This difference is probably due to the presence of other components (e.g. alkalinity) in natural water [1,10,26].

## Conclusions

In this work, RSM was employed for optimization of TOC removal using UV/H_2_O_2_ process. By using the Box-Behnken method, four main parameters including initial TOC concentration, Hydrogen peroxide concentration, pH and time were examined. A second-order polynomial model was developed using multiple linear regression analysis. Statistical test (ANOVA) indicated a good agreement between experimental data and the built model (R^2^ = 0.98). The optimal operating conditions were determined using numerical optimization techniques. For this purpose, the maximum TOC removal was optimized for minimum consumed H_2_O_2_. Accordingly, the removal efficiency of TOC, 78.02% in terms of the independent variables was optimized. Further confirmation experiments under optimum operating conditions showed a 76.50% of TOC removal and confirmed that the model is accordance with the experimental data. The efficiency of TOC removal from natural water based on RSM optimum condition was 62.15%. This study showed that RSM based on Box-Behnken method is a useful tool for optimizing the operating parameters for TOC removal using UV/H_2_O_2_ process.

## Competing interests

The authors declare that they have no competing interests.

## Authors’ contributions

RR has participated in all stages of the study (design of the study, conducting the experiment, analyzing of data and manuscript preparation). AM carried out statistical and technical analysis of data and intellectual helping for analyzing of data. AJ and SM carried out statistical and technical analysis of data, participated in design of study and manuscript preparation. YZ performed data collection and carried out technical analysis. All authors read and approved the final manuscript. AHM participated in the design of the study, final revised of manuscript and intellectual helping for analyzing of data.
